# Exploring the influence of weather variability and climate change on health outcomes in people living with dementia: A scoping review protocol

**DOI:** 10.1371/journal.pone.0304181

**Published:** 2024-06-24

**Authors:** Camila Astolphi Lima, Sara Alsunaidi, Samuel Lowe, David B. Hogan, Liz Dennett, C. Allyson Jones, Shelby Yamamoto

**Affiliations:** 1 School of Public Health, Edmonton Clinic Health Academy, University of Alberta, Edmonton, AB, Canada; 2 Cumming School of Medicine, Departments of Medicine and Community Health Sciences, University of Calgary, Calgary, AB, Canada; 3 Geoffrey and Robyn Sperber Health Sciences Library, University of Alberta, Edmonton, AB, Canada; 4 Department of Physical Therapy, University of Alberta, Edmonton, AB, Canada; University Hospital Cologne: Uniklinik Koln, GERMANY

## Abstract

Environmental factors resulting from climate change and air pollution are risk factors for many chronic conditions including dementia. Although research has shown the impacts of air pollution in terms of cognitive status, less is known about the association between climate change and specific health-related outcomes of older people living with dementia. In response, we outline a scoping review protocol to systematically review the published literature regarding the evidence of climate change, including temperature and weather variability, on health-related quality of life, morbidity, mobility, falls, the utilization of health resources, and mortality among older adults living with dementia. This scoping review will be guided by the framework proposed by Arksey and O’Malley. Electronic search (Medline, Embase, PsycINFO, CINAHL, Scopus, Web of Science) using relevant subject headings and synonyms for two concepts (older people with dementia, weather/ climate change). No publication date or other restrictions will be applied to the search strategy. No language restriction will be applied in order to understand the impact of non-English studies in the literature. Eligible studies must include older adults (65+years) with dementia living in the community and investigate the impacts of climate change and/or weather on their health-related quality of life, morbidity, mobility, falls, use of health resources and mortality. Two independent reviewers will screen abstracts and select those for a full-text review, perform these reviews, select articles for retention, and extract data from them in a standardized manner. This data will then be synthesized and interpreted. OSF registration: DOI: 10.17605/OSF.IO/YRFM8.

## Introduction

The world’s temperature is estimated to increase approximately 1.5°C by 2050 [1], leading to generally negative and potentially irreversible climate consequences [2]. Climate change occurs due to a combination of natural factors and human activities, such as greenhouse gas emissions, deforestation and air pollution. Climate change and air pollution can interact and exacerbate each other given their chemical and physical interactions, common sources, and interconnected environmental effects [3]. The impact of climate change will lead to long-term changes in environments, food security, and population health. Short-term weather changes are projected to include more frequent and severe heat waves as well as more extreme weather events [4].

Studies of the health consequences associated with extreme weather phenomena (e.g., floods and hurricanes) have typically focused on short-term outcomes such as injuries and/or death [5]. Emerging evidence indicates that the long-term effects of climate change can also adversely affect the prevalence, severity, and consequences of respiratory, cardiovascular, and neuropsychiatric diseases [5–8]. The impact of climate change on health does depend on the pre-exposure physical and psychological health of the population as well as social and built environment factors [9]. The rapidly growing older population is potentially vulnerable to extreme weather events due to their reduced physiological reserves [9], pre-existing health conditions, limited mobility, social isolation, and frailty. Older adults typically have a susceptibility to an adverse outcome when exposed to environmental stressors [9–15].

Among vulnerable groups who have reduced adaptive capacity to climate change are older adults living with dementia. Dementia is a prevalent neurocognitive disorder of primarily older adults, which is characterized by cognitive decline, functional limitations [16], and associated mood and behavioural challenges such as apathy, depression, psychotic features, and agitation [17]. With societal aging, the burden of dementia is a public health concern with serious repercussions worldwide, particularly for low to middle income countries [16, 18] and racialized and socioeconomic disadvantaged populations [19]. As dementia is often diagnosed during the middle and later stages of the disease, projections likely underestimate the full impact of this neurocognitive disorder [16]. Alzheimer’s disease (AD) as either the sole cause or a contributing factor (mixed disease) accounts for approximately 60 to 80% of all cases of dementia in older adults. Other common dementias include vascular dementia, dementia with Lewy Bodies, and frontotemporal dementia [20, 21].

Potentially modifiable risk factors for dementia may prevent or delay up to 40% of dementias [22]. These risk factors include: less education, hearing loss, traumatic brain injury, hypertension, alcohol, obesity, smoking, depression, social isolation, physical inactivity, diabetes, and air pollution [22–25]. Initiatives focussing on these risk factors may be effective strategies in delaying or preventing dementia [26, 27]. Health promotion and proactive measures can have significant impacts on the quality of life of this population at all levels of the disease. More advanced stages of dementia are characterized by functional disability requiring assistance with basic activities of daily life [28–30], impaired mobility [31, 32], and a predisposition to falls [33, 34].

Susceptibility to the negative effects of climate change in people living with dementia is related to the stage of disease and associated factors such as co-morbidities and frailty. At advanced stages, vulnerability to climate change manifesting as excessive heat or changing cold patterns can be exacerbated by reduced physiological reserves and the inability to recognize, communicate, and act on hunger or thirst [35], fostering further dependency on caregivers [36]. Medications commonly used by older adults may also adversely affect thermoregulation, alter sweating and urinary output, and/or limit heat perception [37]. People living with dementia are often stigmatized and may be socially isolated [38]. While conceptually different constructs, loneliness [39] and social isolation [40] are risk factors for dementia and are associated with physiologic mechanisms and health behaviours that may precipitate a vulnerability to climate change. There is a complex relationship between environmental factors and older adults living with dementia who are socially isolated and lonely. The difficulty of receiving and comprehending warning information can also further increase their vulnerability [41, 42].

Exposure to air pollution is also a risk factor for dementia [22, 25, 43–45], possibly through its association with cerebrovascular and cardiovascular diseases [46]. In the United States, short-term increases in exposure to fine particles was reflected in a higher risk of hospitalizations and all cause mortality [47]. Fine particles can be directly influenced by meteorological conditions, where higher winds speeds, and rain can facilitate its transportation and dispersion [48, 49].

Heat waves have been associated with increases in the number of emergency department visits, hospitalizations, and deaths in people living with dementia [36, 50, 51]. Little is known about the effects of persistent meteorological exposures such as increased ambient temperature on the cognitive, functional, behavioural (including sleep) and mobility status of people living with dementia. Although the relationship between dementia and the climate have been examined, few studies have specifically addressed the impact on mobility in older adults living with dementia. Other factors such as frailty, social isolation, and functional dependency are associated with reduced mobility that, in turn, contributes to challenges in coping during extreme weather events when evacuation may be required or with seasonal changes leading to, for example, an increased number of falls in winter [15, 52, 53]. Effective public health strategies [54] including the involvement of health professionals to identify at-risk subgroups and disseminating both information and resources can help ameliorate these adverse consequences [5, 13]. Previous reviews of the adverse effects of environmental exposures on the health of those living with dementia have focused on occupational, air pollution, and heavy metal exposures [22, 25, 43, 45, 46]. Growing evidence is examining climate change, whether it is extreme temperatures, extreme weather events or air quality, and its impacts on vulnerable populations such as older adults. While the type of climate event is dependent upon the regional location, a synthesis of the literature is needed to explore what we know regarding the risks of climate change and strategies that might reduce these risks for people living with dementia. This scoping review protocol will describe and synthesize the evidence of climate change, temperature, and weather variability on select health outcomes in older adults living with all-cause dementia.

## Methods

### Protocol design

The scoping review will be conducted according to the methodological framework by Arksey &O’Malley [55]. Based on this framework, we will follow five stages: (1) identifying the research question; (2) identifying relevant studies; (3) selecting studies; (4) charting the data; and (5) collating, summarizing, and reporting the results. The Preferred Reporting Items for Systematic reviews and Meta-Analyses extension for Scoping Reviews (PRISMA-ScR) reported by Tricco et al. [56] will be followed. This scoping review has been submitted to Open Science Framework (DOI: 10.17605/OSF.IO/YRFM8).

### Stage 1: Identifying the research question

The aim of the scoping review is to describe and synthesize literature regarding the specific research question: *What is the evidence of climate change*, *temperature fluctuations*, *and weather variability on the health-related quality of life*, *morbidity*, *mobility*, *falls*, *utilization of health resources*, *and mortality among older adults living with dementia*?

### Stage 2: Identifying relevant studies

The literature search strategy will be conducted in two phases:

- Phase 1: An initial limited search will be conducted on the Medline database developed and implemented by a research librarian with expertise in systematic reviews. The content of the titles, abstracts and index terms will be analyzed. Using this initial search as a guide, the terms will be revised by a team consisting of health professionals, environmental epidemiologists, and a librarian to ensure that relevant keywords are covered in the final search.
- Phase 2: Based on the search findings in Phase 1, the research librarian will refine the electronic search strategy and revise the search for other electronic platforms. The search will be conducted on the following databases: Medline, Embase, PsycINFO, Scopus, CINHAL, and Web of Science. Subject headings and synonyms related to people with dementia and weather/climate change will be used. Details about inclusion criteria are present below (Stage 3 –Study selection).

An example search strategy can be viewed in the S1 File. The reference lists of included articles will be reviewed to capture possible missed articles. Reference lists of relevant reviews will also be checked and any relevant primary studies identified will be manually added.

No language, date of publication or other restrictions will be applied in the literature search. After the electronic search, the references will be uploaded to Covidence®, a web-based software platform for conducting scoping and systematic reviews [57]. The duplicates will be removed by the software and screening will be conducted.

### Stage 3: Study selection

This scoping review is a multidimensional investigation that will consider peer-reviewed research articles that focus on meteorological exposure (weather, climate change) of older adults living with dementia and how these exposures impact their health-related quality of life, morbidity, mobility, falls, use of health resources, and mortality. Studies will be considered eligible according to the criteria described below:

1. Type of study: case-control [58], cohort [59], cross-sectional [58], experimental [58], ecological [60], and qualitative [61] studies will be eligible for this scoping review.
2. Type of population: studies including people with dementia aged 65 years and older living in the community will be eligible. We will follow the World Health Organization definition of dementia, which is a chronic condition that results from different diseases and injuries that affect the brain (primarily or secondarily), altering memory, orientation, comprehension, learning capacity, and judgement [16]. Studies will be included if it is possible to separately extract data about people living with dementia.
3. Exposure: studies that investigate weather or climate change exposure will be considered eligible. We defined weather as a location-based meteorological condition that occurs over a short period of time (e.g., daily fluctuations of temperature, humidity, pressure), and climate change as location-based meteorological conditions over a longer period of time (e.g., average precipitation levels over the last decade in a region) [4].
4. Outcomes: studies must include data on health-related quality of life, morbidity, mobility, falls, use of health resources and/or mortality. Health-related quality of life is defined as “the value assigned to duration of life as modified by impairments, functional states, perceptions, and social opportunities that are influenced by disease, injury, treatment, or policy” [62]. It is recognized that there are challenges in capturing information for those with a dementia especially as dementia severity progresses [63]. Morbidity will be broadly defined as “any departure, subjective or objective, from the state of physiological or psychological wellbeing” [64]. This will include physical and psychological symptoms, physical functioning, role functioning, and overall perception of health [65]. Mobility is defined as the ability to “move by changing body position or location or by transferring from one place to another, by carrying, moving or manipulating objects, by walking, running or climbing, and by using various forms of transportation” [66]. A fall is defined as "as an event which results in a person coming to rest inadvertently on the ground or floor or other lower level" [67]. Both mobility and falls are of particular interest to our group as we suspect they will be sensitive indicators of both risk for harm and adverse effects from climate change and/or weather. The use of health resources (e.g., emergency department visits, hospital admissions, ambulatory visits) are dependent not only on health but also on the availability of resources within the healthcare system.

Studies will be excluded if they:

1. describe health-related impacts that have been linked to weather but do not include older adults living with dementia;
2. include participants who reside in institutions, for example long-term care facilities whose exposures may differ from those living in the community;
3. describe health/mobility related impacts in people living with dementia but without consideration of weather/climate variables;
4. include weather/climate variables, but do not examine or describe the relationship with health/mobility;
5. studies that otherwise meet the inclusion criteria but do not analyze older adults with dementia as a subgroup, limiting the ability to extract relevant data;
6. conference abstracts, book chapters, case reports, theses, dissertations, and letters to the editor will be excluded from this review.

Although this scoping review will not use restrictions related to language, non-English contributions will be excluded from full-text reviews. This is because of our inability to ensure a comparable review of this literature compared to English publications. Studies in languages other than English will be accounted for and described in our flow diagram in order to assess the extent and potential impact of excluding non-English literature from our review.

**Fig 1:**
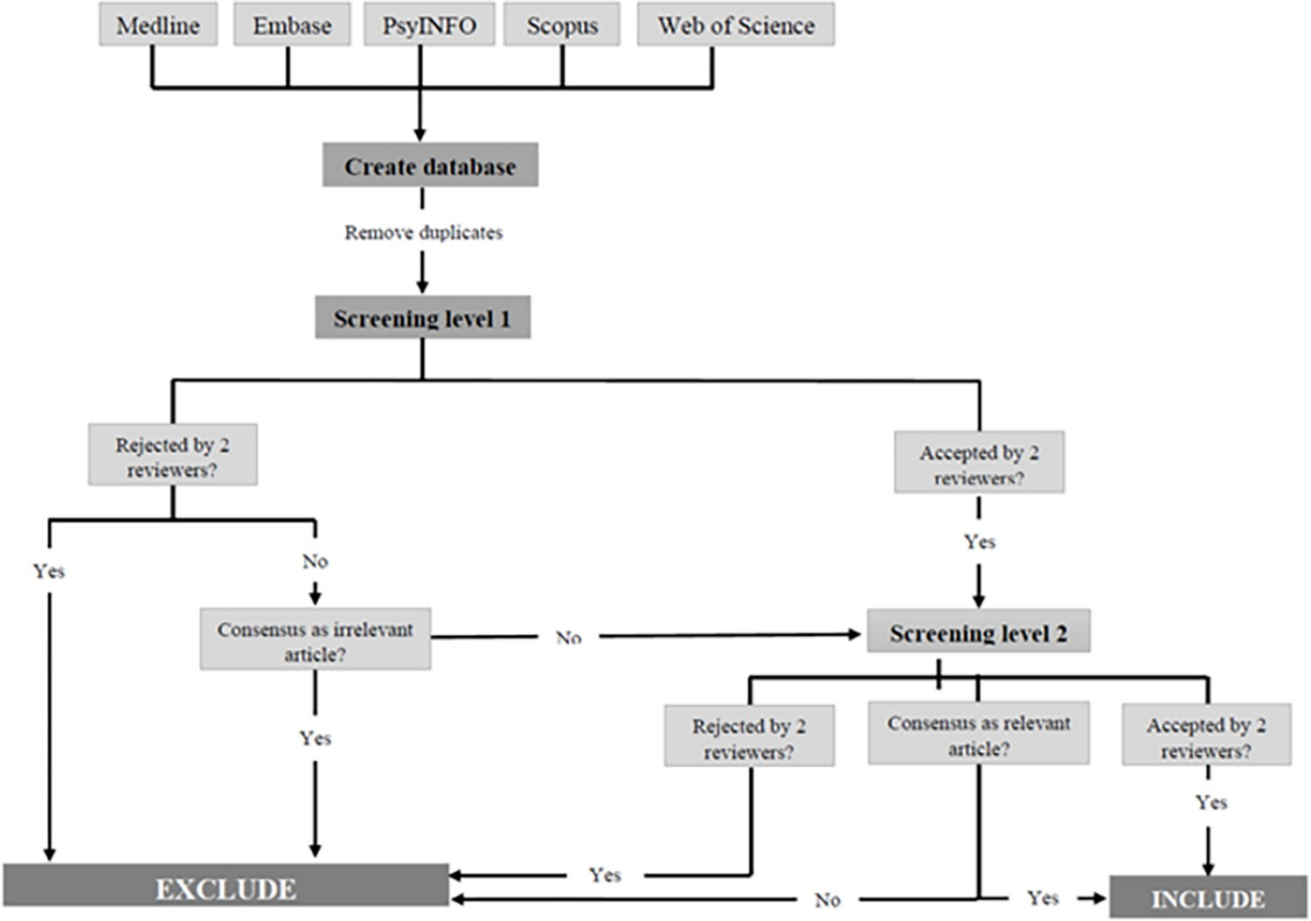
Flowchart from database creation and screening selection.

Screening will be conducted in two phases. Detailed steps of the screening process are presented in Fig 1. Two independent reviewers will determine the eligibility of the articles at screening phases. The reviewers will screen all titles and abstracts captured in the search. A structured form (S2 File) containing the inclusion and exclusion criteria will be used as a guide. The reviewers will screen the first 50 articles to determine the level of consensus [56]. The team will also discuss possible adjustments to the protocol as well as the screening form guide. A Cohen’s K statistic will be generated to determine inter-rater agreement [68]. After 80% agreement is achieved, the first screening phase will start. Studies considered relevant will be included in phase two. At the second phase, reviewers will use a structured form (S2 File) to screen full-text articles. Like the abstract screening stage, five articles will be screened initially to optimize consensus. Possible adjustments will be discussed and, if necessary, adjustments will be undertaken. Once 80% agreement between the 2 reviewers is achieved, the full text screening phase will start. Only articles that meet all inclusion criteria will be included. Reviewers will meet regularly to discuss any disagreements, and to consult a third reviewer to reach consensus as necessary. The team also will meet regularly to update the review guides and form.

### Stage 4: Charting the data

A standardized form will be developed for the data extraction phase. This form will be piloted by the reviewers to determine the quality of extracted data and to identify possible issues. The team will discuss the findings, and the form and tables will be revised accordingly.

Data extraction will then be conducted independently by two reviewers. In the case of disagreement, a third reviewer will be consulted to reach consensus. Data regarding each article (author, title, year of publication, journal, country, funding source), study characteristics (objectives, study design and methods, setting [e.g., community], duration of follow-up), participants’ characteristics (number, age, sex, type, and severity of dementia), exposure characteristics (type of weather/climate exposure, assessment methods) and main outcomes (health-related quality of life, morbidity, mobility, falls, use of health resources, and mortality) will be extracted (S1 Table). In the case of incomplete or unclear data, the original authors will be contacted. If they do not answer or cannot provide what is requested, the information will be considered as missing.

### Stage 5: Collating, summarizing, and reporting the results

Studies will be first categorized by type of exposure (e.g., floods, drought, wildfires, storms, extreme temperature, average ambient temperature rise). Outcomes will then be categorized by health-related quality of life, morbidity, mobility, falls, and use of health resources (e.g., hospitalizations, emergency room visits) and mortality. The results will be reported descriptively, using frequencies and percentages. The outcomes will be presented separately for each type of exposure in tabular format. To facilitate clarity, a figure summarizing the main outcomes will be generated [69].

### Ethics and dissemination

Ethics will not be required in this study because we will use only publicly accessible documents. The dissemination plan for this study includes publishing findings in a peer reviewed journal, presenting them at relevant conferences, and digital resources. Short videos summarizing our findings will be created to post on websites and social media. Also, we will include our results as a point of discussion in relevant podcasts and news articles.

## Discussion

There are three possible limitations of this scoping review: underestimation of dementia diagnoses, study design of the included studies, and studies from different countries.

There is a misconception that dementia is a result of getting old, leading to an underestimating diagnosis and, consequently, making even more difficult estimate the real number of people living with dementia worldwide [70]. This fact becomes a limitation when conducting studies in this population, since the results are underestimated.

We believe that, for the most part, selected studies will be an ecological design. These studies have peculiar characteristics that can lead to a risk of bias, for example, ecological fallacy, and prevent the drawing of causal relationships. Although our scoping review will not include a risk of bias analysis, it is important consider the level of evidence in these syntheses.

This scoping review is not restricted to a particular country or region; therefore, we will be able to identify relevant data regarding the effects of climate change and weather on people living with dementia.

In spite of these limitations, we will be able to provide a synthesis of the currently available evidence to contribute to our understanding of the implications of climate change on the health of people living with dementia and highlight gaps in the literature.

Specifically, our aim is to synthesize the published literature to generate an overview of the impact of climate change and the health-related quality of life, morbidity, mobility, falls, use of health resources and mortality in older adults living with dementia. This review will also identify data and knowledge gaps that need to be addressed. Public health strategies are integral in dealing with climate change impacts on vulnerable populations such as people living with dementia. A first step is to synthesize the evidence, which can then inform further research and public health measures such as surveillance that are inclusive of such populations.

## Supporting information

S1 Checklist PRISMA checklist. (PDF)

S1 File Preliminary search strategy. (PDF)

S2 File Draft screening structured form. (PDF)

S1 Table Draft data extraction tables. (PDF)
