# Supplementary material for: Exploring the influence of weather variability and climate change on health outcomes in people living with dementia: A scoping review protocol
Source: PLoS One. 2024 Jun 24;19(6):e0304181. doi: 10.1371/journal.pone.0304181 (PMC11195938; doi:10.1371/journal.pone.0304181)
Supplement: S1 File — (PDF) [file pone.0304181.s002.pdf]

Supporting Information 1

Preliminary search strategy

Database: Ovid Medline

1. weather/ or climate/ or climatic processes/ or exp climate change/ or cyclonic storms/ or droughts/ or floods/ or greenhouse effect/
2. (temperature adj10 (annual* or daily or month* or ambient or season* or climate or climatic or weather or summer or winter)).mp.
3. Seasons/
4. extreme heat/ or hot temperature/
5. cold temperature/ or freezing/
6. (((climat* not (political-climate or organi?ational-climate or economic-climate or financial-climate)) or weather) adj3 (chang* or disrupt* or volati* or instabilit* or unstable or variable or variability or vulnerab*)).mp.
7. ((chang* or decline* or decrease* or increase*) adj3 humidity).mp.
8. ((global* or climate) adj2 warm*).mp.
9. ((climat* or weather) adj (conditions or factors or driven)).mp.
10. (greenhouse gas* or greenhouse effect* or carbon emission* or carbon dioxide emission* or CO2 emission*).mp.
11. (((extreme or severe) adj (weather or heat or cold or temperature*)) or hot-weather or heat-wave* or heatwave or high-temperatures or freezing-temperature* or low-temperatures or ((higher or lower or cold* or warm* or hot*) adj3 temperature*)).mp.
12. ((annual* or daily or month* or ambient or environmental or season* or climate or climatic or weather or summer or winter) adj4 temperature*).mp.

13. (warm-season* or wet season* or dry season* or cold season* or warm*-month* or (unseasonabl* adj3 (warm or hot or heat or humid* or cold or high or low))).mp.

14. (El Nino or la nina).mp.

15. (rain or rains or rainstorm* or raining or rainfall or (snow not visual snow) or snows or snowy or snowfall* or icy or ((high or heavy or extreme or severe or daily or levels) adj2 precipitation) or UV-index or flooding or floods or waterlogging or (drought* not drought-resistan*) or desertifcation or hurricane* or cyclone* or tornado* or superstorm* or dust storm or storm surge* or ice storm* or ((storm or storms) and disaster*) or monsoon*).mp.

16. (wildfire* or fire disaster* or (uncontrolled adj3 fire*) or forestfire* or bushfire* or ((wild* or bush or forest* or vegetation or landscape) adj6 fire*)).mp.

17. famine*.mp.

18. 1 or 2 or 3 or 4 or 5 or 6 or 7 or 8 or 9 or 10 or 11 or 12 or 13 or 14 or 15 or 16 or 17

19. dementia/ or alzheimer disease/ or exp aphasia, primary progressive/ or exp dementia, vascular/ or exp frontotemporal lobar degeneration/ or huntington disease/ or lewy body disease/

20. lewy body disease/ or parkinson disease/

21. (dementia or pick* disease or cadasil or (parkinson* not parkinsonia*) or alzheimer* or huntington* or lewy body or cognitive* impair* or neurocognitive disorder* or intellectual*-impair* or cognitive-dysfunction or cogniti*-disorder* or primary-progressive-aphasia).mp.

22. neurocognitive disorders/ or cognition disorders/ or cognitive dysfunction/

23. 19 or 20 or 21

24. or/19-23

25. 18 and 23

26. 18 and 24

27. limit 26 to animals

28. limit 27 to humans

29. 26 not (27 not 28)

30. (mice or mouse or rat or rats or rodent* or animal model or zebrafish*).ti.

31. 29 not 30
