# Supplementary material for: Exploring the influence of weather variability and climate change on health outcomes in people living with dementia: A scoping review protocol
Source: PLoS One. 2024 Jun 24;19(6):e0304181. doi: 10.1371/journal.pone.0304181 (PMC11195938; doi:10.1371/journal.pone.0304181)
Supplement: S2 File — (PDF) [file pone.0304181.s003.pdf]

Supporting Information 2

Draft screening structured form

Level 1 – Title and abstract screening

- Is it a case-control, cohort, cross-sectional, clinical trials, epidemiological, ecological, intervention, or review article?

  ☐ Yes ☐ No ☐ Maybe

- Does the research focus on weather (e.g., wildfires, floods, extreme temperatures, icy conditions, heat, or cold waves) or climate change?

  ☐ Yes ☐ No ☐ Maybe

- Does the research focus on health/mobility (including falls) impacts of weather?

  ☐ Yes ☐ No ☐ Maybe

- Does the research include people with dementia?

  ☐ Yes ☐ No ☐ Maybe

If 'yes' or 'unsure' is selected, study will be moved onto the next stage.

Level 2 – Full-text screening

- Does the research focus on weather or climate change?

  ☐ Yes ☐ No

- Does the research focus on health/mobility (including falls) impacts of weather?

  ☐ Yes ☐ No

- Does the research focus on older adults (at least 80% of included participants 65+ - except for studies about mortality and emergency admissions)?

  ☐ Yes ☐ No

- Does the research focus on people who reported or have diagnoses of dementia (at least 80% of included participants - except for studies about mortality and emergency admissions)?

  ☐ Yes ☐ No
