# Supplementary material for: Exploring the influence of weather variability and climate change on health outcomes in people living with dementia: A scoping review protocol
Source: PLoS One. 2024 Jun 24;19(6):e0304181. doi: 10.1371/journal.pone.0304181 (PMC11195938; doi:10.1371/journal.pone.0304181)
Supplement: S1 Table — (PDF) [file pone.0304181.s004.pdf]

Supporting Information 3

Draft data extraction tables

Section	Question	Data
**Study information**	Reviewer	*Name*
	Authors	*Answer*
	Title	*Answer*
	Published Year	*Answer*
	Journal	*Answer*
	Country	*Answer*
**Study characteristics**	What was the study objective(s)?	*Answer*
	What is the study design and methods?	*Answer*
	What was the setting (e.g., community)?	*Answer*
	In which city/country was the data collected?	*City, Country*
	Was any database used for data collection?	*Answer*
	When was the data collected (start and stop dates)?	*Month, Year*
**Sample characteristics**	What was the total number of participants/ participants per group?	*Answer*
	What was the age range?	*Answer*
	What was the sex distribution?	*Answer*
	Did they report any comorbidity besides dementia?	*Answer*
	Did they report on medications being taken?	*Answer*
**Dementia characteristics**	What was the number/ proportion of participants with dementia?	*Answer*
	What was the type of dementia?	*Answer*

	How long time since the diagnosis (in months)?	*Answer*
	How was dementia diagnosed?	*Answer*
	Which cognitive screening tool was used?	*Answer*
**Outcomes**	What climate/weather variables were investigated?	*Answer*
	How were climate/weather variables defined?	*Answer*
	How were climate/weather variables measured?	*Answer*
	What were the outcomes analysed?	*Answer*
	How were the outcomes measured?	*Answer*
	What was the follow-up time?	*Answer*
	What are the main results related to health-related quality of life (in summary)?	*Answer*
	What are the main results related to morbidities (in summary)?	*Answer*
	What are the main results related to use of health resources (in summary)?	*Answer*
	What are the main results related to mortality (in summary)?	*Answer*
	What type of statistical analysis was used?	*Answer*
**Conclusion**	What were the relevant conclusions of the study?	*Answer*
